# Outstanding Reviewers for *RSC Advances* in 2021

**DOI:** 10.1039/d2ra90051c

**Published:** 2022-07-26

## Abstract

We would like to take this opportunity to highlight the Outstanding Reviewers for *RSC Advances* in 2021, as selected by the editorial team for their significant contribution to the journal.
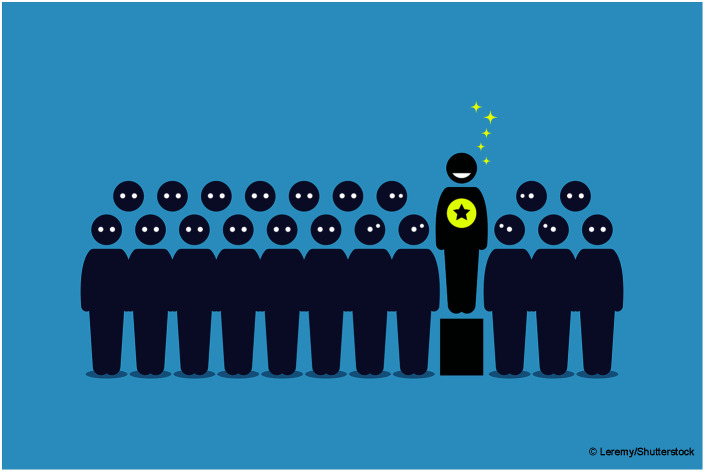

We would like to take this opportunity to thank all of *RSC Advances*’s reviewers, and in particular highlight the Outstanding Reviewers for the journal in 2021, as selected by the editorial team for their significant contribution to *RSC Advances*. We announce our Outstanding Reviewers annually and each receives a certificate to give recognition for their contribution. The reviewers have been chosen based on the number, timeliness and quality of the reports completed over the last 12 months.

 

Dr Gopi Adhikari

University of Nebraska-Lincoln

ORCID: 0000-0002-9986-8218

 

Dr Marco Anni

Università del Salento

ORCID: 0000-0002-1651-0166

 

Dr Jubaraj Baruah

Indian Institute of Technology Guwahati

ORCID: 0000-0003-3371-7529

 

Dr Mauro Chinappi

Università degli Studi di Roma Tor Vergata

ORCID: 0000-0002-4509-1247

 

Professor Søren Christensen

Københavns Universitet

ORCID: 0000-0002-5773-6874

 

Professor Beelee Chua

Korea University

ORCID: 0000-0002-9153-0167

 

Dr Francesco Ferlin

Università degli Studi di Perugia

ORCID: 0000-0003-3800-9708

 

Dr Lihua Gan

Tongji University

 

Dr Charles Gauthier

Institut National de la Recherche Scientifique

ORCID: 0000-0002-2475-2050

 

Dr Xuyun Guo

The Hong Kong Polytechnic University

ORCID: 0000-0003-0365-7545

 

Professor Wei-Min He

University of South China

ORCID: 0000-0002-9481-6697

 

Dr Bolong Huang

The Hong Kong Polytechnic University

ORCID: 0000-0002-2526-2002

 

Professor Dong-Hau Kuo

National Taiwan University of Science and Technology

ORCID: 0000-0001-9300-8551

 

Dr Dattatray Late

National Chemical Laboratory CSIR

ORCID: 0000-0003-3007-7220

 

Professor Giuseppe Lazzara

Università degli Studi di Palermo

ORCID: 0000-0003-1953-5817

 

Dr Xin Liu

University of Florida

ORCID: 0000-0001-9504-795X

 

Dr Nadia Mahmoud Tawfiq Jebril

University of Plymouth

ORCID: 0000-0002-5368-2127

 

Dr Ramakanta Naik

Institute of Chemical Technology Mumbai - IndianOil Odisha Campus Bhubaneswar

ORCID: 0000-0002-4460-1540

 

Dr Yangguang Ou

University of Vermont

ORCID: 0000-0002-6902-3978

 

Dr Paresh Samantaray

Indian Institute of Science

ORCID: 0000-0003-2533-929X

 

Dr Dane Scott

East Tennessee State University College of Arts and Sciences

ORCID: 0000-0003-0018-7189

 

Dr Rodolfo Teixeira

University of Nottingham

ORCID: 0000-0001-8042-8442

 

Dr Carlos Torres-Torres

Instituto Politécnico Nacional

ORCID: 0000-0001-9255-2416

 

Dr Renjie Wang

Virginia Commonwealth University

ORCID: 0000-0002-2969-0987

 

Dr Zhixin Wang

University of Florida

ORCID: 0000-0001-7255-6049

 

Dr Biquan Xiong

Hunan Institute of Science and Technology

ORCID: 0000-0002-6490-6384

 

Dr Li-Ming Yang

Huazhong University of Science and Technology

ORCID: 0000-0002-7836-212X

 

Dr Zhi Yue

The University of Chicago

ORCID: 0000-0002-4231-7474

 

Dr Wen Zhang

Tianjin University

ORCID: 0000-0001-6118-3136

 

Professor Guowei Zhou

Qilu University of Technology

ORCID: 0000-0002-7023-6225

 

We would also like to thank the *RSC Advances* Editorial Board and Advisory Board and the research community for their continued support of the journal, as authors, reviewers and readers.

 

Russell Cox, Editor-in-Chief

Laura Fisher, Executive Editor

## Supplementary Material

